# Wearable and battery‐free wound dressing system for wireless and early sepsis diagnosis

**DOI:** 10.1002/btm2.10445

**Published:** 2023-02-01

**Authors:** Jiyu Li, Xingcan Huang, Yawen Yang, Jingkun Zhou, Kuanming Yao, Jian Li, Yingying Zhou, Meixi Li, Tsz Hung Wong, Xinge Yu

**Affiliations:** ^1^ Department of Biomedical Engineering City University of Hong Kong Kowloon Tong Hong Kong; ^2^ Hong Kong Center for Cerebra‐Cardiovascular Health Engineering Hong Kong Science Park New Territories Hong Kong; ^3^ Department of Biomedical Engineering Hong Kong Polytechnic University Kowloon Hong Kong; ^4^ Leshan Hospital of Traditional Chinese Medicine Leshan China

**Keywords:** battery‐free electronics, bio‐integrated electronics, biosensors, sepsis, wound dressing

## Abstract

Sepsis is a severe organ dysfunction typically caused by wound infection which leads to septic shock, organ failure or even death if no early diagnosis and property medical treatment were taken. Herein, we report a soft, wearable and battery‐free wound dressing system (WDS) for wireless and real‐time monitoring of wound condition and sepsis‐related biomarker (procalcitonin [PCT]) in wound exudate for early sepsis detection. The battery‐free WDS powered by near‐field communication enables wireless data transmission, signal processing and power supply, which allows portable intelligent wound caring. The exudate collection associates with soft silicone based microfluidic technologies (exudate collection time within 15 s), that can filtrate contamination at the cell level and enable a superior filtration rate up to 95% with adopting microsphere structures. The battery‐free WDS also includes state‐of‐the‐art biosensors, which can accurate detect the pH value, wound temperature, and PCT level and thus for sepsis diagnosis. In vivo studies of SD rats prove the capability of the WDS for continuously monitoring wound condition and PCT concentration in the exudate. As a result, the reported fully integrated WDS provides a potential solution for further developing wearable, multifunctional and on‐site disease diagnosis.

## 1 INTRODUCTION

Sepsis associating with a complex and extreme immune response to pathogens‐induced infection is a potentially life‐threatening disease with high levels of morbidity and mortality.^1^ Without timely diagnosis and treatment, sepsis can rapidly result in organ damage, immune response disorder and even death.^2^, ^3^, ^4^ Procalcitonin (PCT), a member of the calcitonin super family, is a critical biomarker for the septic disease diagnosis where level of PCT in the serum increases drastically when sepsis happens.^5^ Current clinical relevant testing of PCT associates with the measurement of PCT concentration in the serum or plasma collected from the patient,^6^, ^7^ which is inconvenient, time consuming, and require medical care personnel. Infection of wound is a key reason to cause sepsis which is commonly caused by physical injury, chemical damages, and operation surgery.^8^, ^9^ The wound exudate derived from blood contains a series of biophysical, biochemical, and pathological information of septic disease such as PCT, Ph value, and so forth.^10^ In addition, wound can also cause skin temperature increasing of skin near the lesion area.^11^ In real‐time monitoring of disease biomarkers from wound related exudate allow to achieve the point of care test on skin, and thus provides potential application in septic disease diagnosis.

Over the past decade, flexible electronics integrated wound dressings have been developed for wound monitoring and management.^12^, ^13^ The advances in functional materials development and microfabrication technologies enable developing novel intelligent system for wearable wound exudate management with the capabilities of real‐time monitoring and data transmission through cutting‐edge wound dressing system (WDS).^14^, ^15^, ^16^ However, none of the WDSs are reported for sepsis diagnosis based on wound monitoring. The current WDS is typically restricted in accuracy detection of PCT from exudate for septic disease monitoring due to contamination and limited collection capacity of exudate.^17^ In addition, the PCT level in wound exudate is significantly lower than serum due to the dilution effect.^18^ Therefore, developing advanced WDS with the capacity of anti‐contamination, high exudate collection efficiency for real‐time monitoring of sepsis relative biomarker in wound condition will provide a new possibility in septic disease diagnosis.

To realize the aforementioned intelligent WDS, there are two key factors need to be considered, where the first one associates with bio‐fluid collection and the other one is accurate sensing of biomarkers. Regarding to the bio‐fluid collection issue, microfluidics techniques have been widely recognized as useful and powerful tools for manipulation of fluidics.^19^, ^20^, ^21^ With the advantage of lower reagent consumptions, high throughput, and easily to carry, the microfluidic device has been largely applied in biomedical engineering,^22^, ^23^ clinical diagnosis,^24^, ^25^ and biology research.^26^, ^27^ Meanwhile, microfilter consisting of micropillar array in the microfluidic device can be applied for the filtration of impurity in the fluidic. Another point of accurate sensing technologies, the recent advances of skin‐mounted biosensors have built strong foundations for on skin bio‐sensing.^28^, ^29^ Thus, the WDS with the integration of microfluidic device and soft biosensors will improve the exudate collection capacity, anti‐contamination, and accurate sensing capabilities during the monitoring of wound condition.

Here, we report a soft, skin‐integrated, and battery‐free WDS for wireless wound monitoring and exudate management for sepsis diagnosis. The WDS consists of the following key parts, a microfluidic module, a wound sensing patch and a flexible printing circuit board (fPCB) with relative electronic components. The WDS could be directly worn on human body seamlessly for exudate collection and skin temperature sensing. The microfilter‐based microfluidic module applied on the wound could filtrate the contaminants in exudate and reduce the sample consumptions (10 μl of exudate sample for one testing). The exudate guided by microfluidic module can be fulfilled into the chamber within 1 min. The PCT sensor and pH sensor with the sensitivity of 1.53 μA and 41.9 mV per decade offers accurate signal acquisition of PCT and pH value in wound exudate. The wireless communication method based on near‐field communication (NFC) is applied for signal processing, data transmission and wireless power supply from portable devices such as smartphones. We systematically investigate the performance of the WDS on wound monitoring in vivo (rat sepsis model). The WDS enables a timely monitoring of PCT and pH values in the exudate secreted from septic rats. In addition, the temperature variation of septic rat could be recorded through temperature sensor in the WDS. Thus, with the efficient exudate collection capacity and sensitive detection of septic relative biomarkers, the WDS exhibit great potential for a common and useful tool in real‐time and point of care monitoring for protein‐based biomarkers in exudate, especially for pathogen‐induced infection, immune system disease, sepsis, and so on.

## 2 RESULTS

### 2.1 Design and characterization of the WDS

**FIGURE 1:**
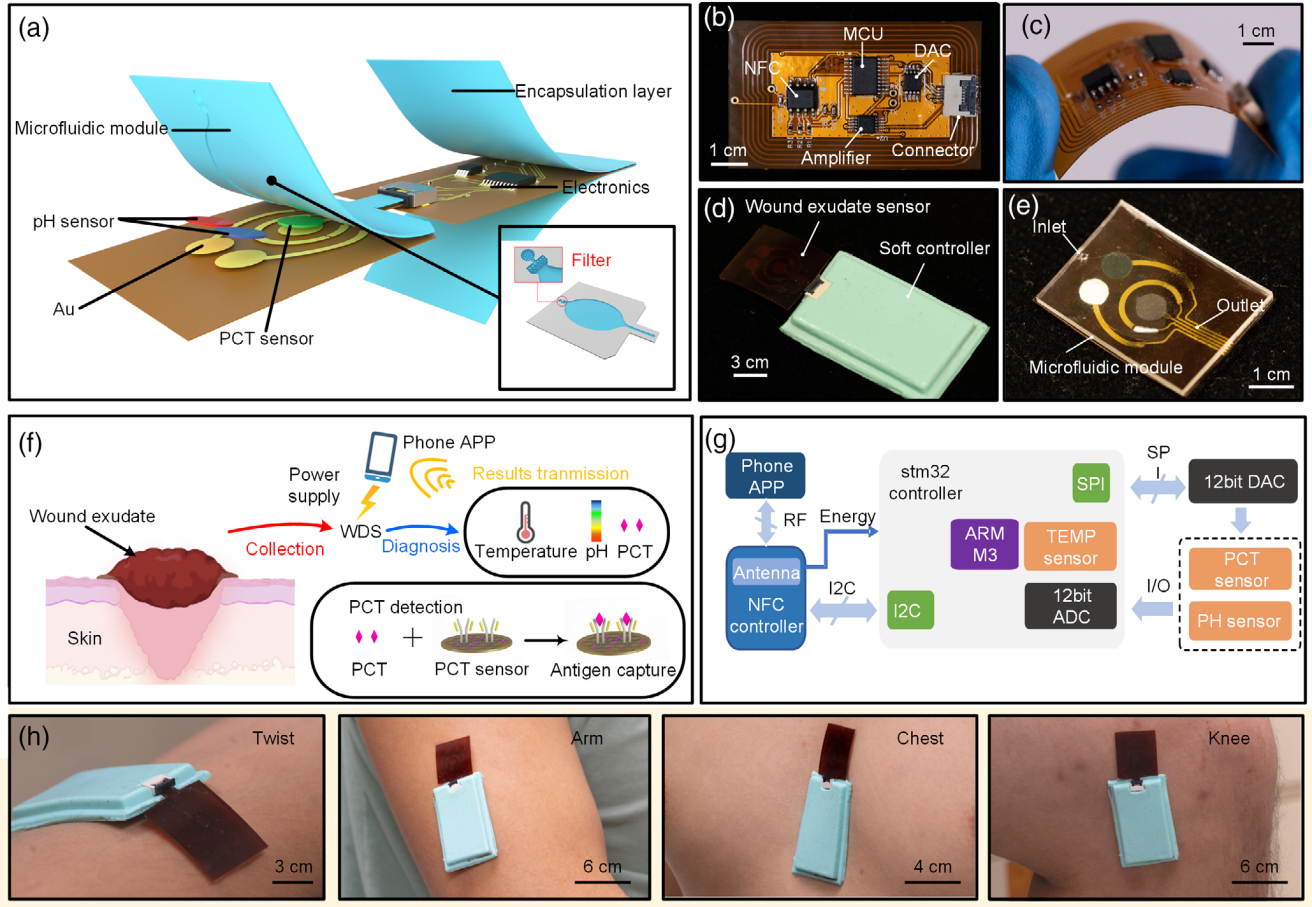
Schematic illustration of the wearable and battery‐free wound dressing system (WDS). (a) Explosive view of the components and assembly method of the WDS. (b–e) Photograph of near‐field communication (NFC)‐based flexible printed circuit board (b), the whole system exhibits great flexibility under bending (c). (d) The optical image of flexible printing circuit board (fPCB) with the integration of Eco‐flex encapsulation layer combined with wound exudate sensor using connector (e). (f) The mechanism of WDS applied on wound for simultaneous monitoring of temperature, pH value, and procalcitonin (PCT). The smart phone is used to wireless power supply, data processing, and transmission. (g) Block diagram and working principle of the wound dressing system. (h) The photograph of WDS applied on the twist, arm, chest, and knee.

Figure 1a shows the overview schematic illustration of the wearable and battery‐free WDS, which consists of a microfluidic chip for filtration and collection of wound exudates, a wound monitoring patch with the integration of a pH sensor and a PCT sensor for pH and biomarkers detection, and a reusable NFC embedded fPCB for signal processing, temperature sensing, wireless data transmission and power management. The optical image of the fPCB is shown in Figure 1b. Without the limitation of internal batteries, the circuit board can be ultrathin and thus exhibits excellent flexibility (Figure 1c and Supplementary Figure 1). To reduce allergic reaction from external stimuli, a thin Ecoflex layer with a thickness of 5 mm was applied as an encapsulation layer on both side of the fPCB (Figure 1d). Moreover, the polydimethylsiloxane (PDMS)‐based microfluidic chip enable strong bonding with the substrate (polyimide [PI]) of the wound exudate sensor by covalent bonding, which can avoid sample leakage during the exudate sensing (Figure 1e). The optical image of the entire device shows that the WDS exhibits an excellent flexibility which can interface with various body parts such as twits, arm chests, and even knees (Figure 1h). The intrinsically soft nature of the WDS offers a high possibility to integrate with commercial dressing for wound healing, waterproof, and ventilation. The monitoring mechanism of the WDS applied on wound is illustrated in Figure 1f. When sepsis happens due to immune system overactions, secretion of the PCT level in blood would increase dramatically. The PCT proteins transport through blood circulation and seep into wound from blood capillary.^30^ After filtration and collection through the microfluidic chip, PCT in the wound exudate can be captured by the PCT antibodies which leads to a variation of surface impedance in the sensor. Meanwhile, the change of the pH value and temperature would also be recorded by the WDS and wireless transmitted for real‐time sepsis assessment.

### 2.2 Circuit designs and operating principles

Figure 1g shows the working principle of the wireless and battery‐free WDS. Briefly, the NFC antenna is applied for wireless power harvesting and data transmission to the smartphone. The system on a chip microcontroller (MCU) is used for data processing, acquisition and storage from the wound monitoring patch. The analog signal outputs collected from the sensors can be converted into digital signals by a 12‐bit analog‐to‐digital converter (ADC) then transmitted to the MCU. Finally, the transmitted data are transferred to the smartphone by the NFC antenna when the smartphone approached.

During the WDS working process, the integrated sensing patch are directly attached onto the skin and connect with the fPCB by an FFC connector. The power can be derived from the energy‐harvesting feature of the NFC when a smartphone gets close to the fPCB, where the whole circuit can be activated simultaneously. Measurements of PCT concentration, pH value, and temperature associate with the variations of differential pulse voltammetry (DPV), open‐circuit voltage (OCV), and integral temperature sensor (Supplementary Tables 1 and 2) of the MCU, respectively. The circuit of the DPV is virtually a miniature and portable potent state in the three‐electrode electrochemical, which realizes a constant potential pole by controlling electrode potential, and thus can be used in the rapid detection of PCT potency. The circuit of the potential measurements incorporates with bias circuit and low‐pass filter, which achieves the open circuit of ion electrode. The analog signals from these circuits then go through the ADC inside the MCU to convert into digital signals. When the result of sensors delivered form MCU via inter‐integrated circuit, the chip of the NFC can transfer data to the phone via RF interfaces.

### 2.3 Microfluidic system design and operation

**FIGURE 2:**
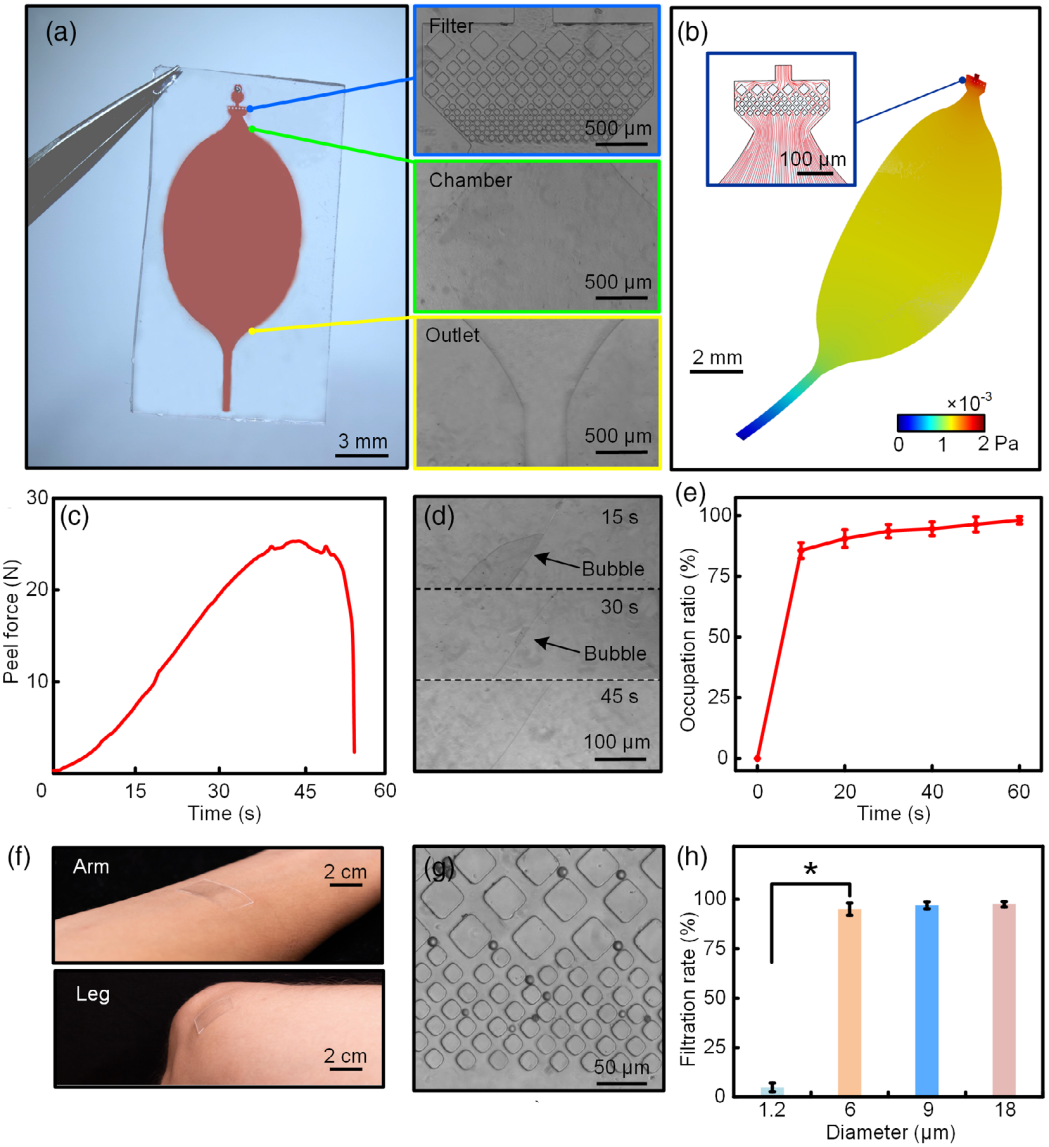
The investigation of the performance of microfluidic chip for exudate collection and filtration. (a) Optical image of polydimethylsiloxane (PDMS)‐based microfluidic system, the micrograph shows the microfilter, monitoring chamber, and outlet in the microfluidic system. (b) Simulated distribution of driving pressure in microfluidic system. The inset shows the simulated velocity profiling and streamline near the inlet (red). (c) The peel force variation between the microfluidic system and wound sensing patch (peeling rate: 2 mm/min). (d) Snapshot of exudate collection and air degassing inside chamber. (e) Occupation ratio for exudate flow into microfluidic chamber along with the time (*n* = 3). (f) Photograph of microfluidic chip applied seamlessly on the arm and leg. (g) The optical image of microsphere with various of diameter filtrated by microfilter. (h) Filtration rate for microsphere with different diameters went through the microfluidic device (*n* = 3).

In the design of the microfluidic system, a microfluidic system serves as the wound exudate collection and filtration (Figure 2a). The microfluidic system contains two parts, the microfilter and oval shape microchamber. The microfilter is integrated with a well‐arranged micropillar array with three different side length (50, 35, and 20 μm, height: 50 μm) for wound exudate filtration. These micropillars allow to form three isolation gaps with distances of 35, 20, and 5 μm connected to the inlet (diameter of 0.5 mm, chamber volume of 10 μl) as microfilter. The oval shape microchamber connected with the microfilter is applied for biosignal monitoring. The exudate secreted from wound can be easily filtrated and “guided” into the oval monitoring chamber by hydraulic pressure even under bending or twisting states (results shown in the computational results by COMSOL Multiphysics, Figure 2b). The great flexibility of the soft PDMS‐based microfluidic chip enables effective exudate collection on different body parts (Figure 2f). The streamline profiling of the exudate going through microfiltration shows a homogeneous distribution, allowing for a similar rate of sweat fluid flow from the wound into the microchamber. At the same time, the synergistic effect between microfluidic chip and wound monitoring patch also plays a key role in wound exudate monitoring. The nearly 25 N peel force between PDMS‐based microchip together with the sensing patch through covalently binding enable a reduction of sample loss due during monitoring (Figure 2c and Supplementary Figure 2). In addition, sample collection is also critical. After processing hydrophilic surface modification of the microchamber via the deposition of polyvinyl alcohol (PVA), the fluid can fill up to 80% of the area in microchamber within 15 s and fulfilled the chamber in 1 min (Figure 2e). The 3D velocity profiling results of the exudate go through into the chamber indicates the sweat fluid always flows along the center of the oval chamber without shifting. Also, the oval shape design of the microchamber facilitates air degassing which restrict the formation of “dead space,” leading to more accuracy signal acquisition (Figure 2d and Supplementary Figure 3). Therefore, this microfluidic system exhibits good efficiency in sample collection.

For the exudate filtration, we first mixed microspheres with various diameter (1.2, 6, 9, and 18 μm) in distilled water as a sample for investigation of the microsphere isolation rate. As shown in Figure 2g, the microfluidic chip together with the microfilter enable a great filtration performance of the microspheres. Nearly 96% microspheres with a diameter >5 μm can be filtrated, which indicates the microfluidic system can provide excellent performance of skin debris filtration during the exudate collection (Figure 2h). Therefore, the PDMS‐based microfluidic chip provides an efficient sample collection rate and reduces skin debris interruption.

### 2.4 Assessment of the biosensor for wound exudate monitoring

**FIGURE 3:**
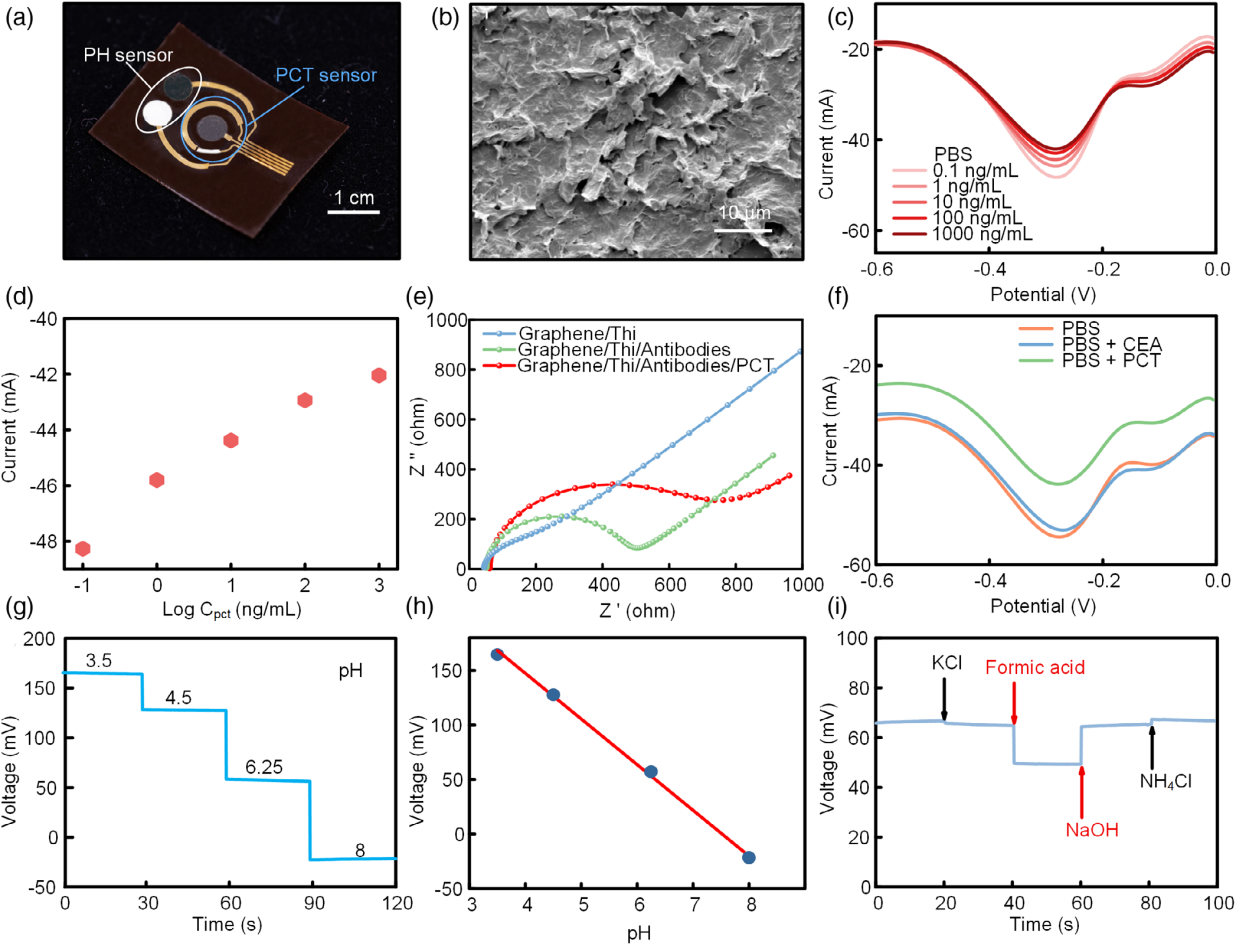
Characterization of the integrated biosensor patch. (a) Optical image of the integrated biosensor patch. (b) SEM image of the sprayed graphene/Thi film on the procalcitonin (PCT) sensor. (c, d) Differential pulse voltammetry curve of the PCT sensor and the corresponding calibration curve. (e) Electrochemical impedance spectroscopy of the graphene/Thi. Graphene/Thi/antibodies and graphene/Thi/antibodies/PCT electrodes. (f) Anti‐interference characterization of the PCT sensor. (g, h) Open‐circuit voltage‐time curve and the corresponding calibration curve of the pH sensor. (i) Anti‐interference characterization of the pH sensor.

Figure 3 shows the results of the characterization of the integrated bio‐sensing patch for real‐time monitoring pH value and PCT concentration in the wound‐induced fluid. As shown in Figure 3a, the state‐of‐the‐art pH sensor and PCT sensor are integrated on a thin, flexible PI substrate, which are able to achieve the in situ monitoring of pH and PCT concentration. Here, the PCT antibodies immobilized on the working electrode are applied to capture the PCT in wound tissue fluids, since the bonding of PCT could increase the surface impedance of the electrode. In order to improve the immobilized amounts of the antibodies and the performance of the PCT sensor, a mixing solution of graphene/thionine (Thi) was uniformly spayed on the gold electrode to serve as the supporting material for PCT antibodies (Figure 3b). As a result, the PCT sensor exhibits excellent sensitivity and good linearity for PCT, as exhibited in Figure 3c,d, where the DPV current would decrease with the increase of the PCT concentration with a coefficient of determination (*R* ^2^) of 0.984 and a sensitivity of 1.53 μA per decade. To further evaluating the performance of the PCT sensor, electrochemical impedance spectroscopy curves of various electrodes are measured (Figure 3e). As expected, the impedance (diameter of the Nyquist curve) of the graphene/Thi/antibodies electrode is greater than that of the graphene/Thi electrode, demonstrating that more antibodies are immobilized on the electrode of graphene/Thi/antibodies. Similarly, the graphene/Thi/antibodies/PCT electrode shows the largest impedance and great combination abilities for PCT, which are the main reasons for decreasing DPV current. Besides, the PCT shows the excellent anti‐interference ability for other antigens. As shown in Figure 3f, the decrease of the DPV current occurs only the PCT added into the electrolyte, while the addition of other antigens (like carcinoembryonic antigen) has negligible effects on the DPV current, benefiting from the perfect specificity of the PCT antibodies. The pH sensor adopts gold electrodes with polyaniline (PANI) electrodeposited on as the sensing part, which exhibits hydrogen ion (H^+^) sensing ability because of its high conductivity in the acid solution.^31^, ^32^ Figure 3g,h shows the OCV responses of the pH sensor and the corresponding calibration curve, respectively. As expected, with the increase of the pH values, the conductivity of the PANI would get worse with the decrease of H^+^ concentration in electrolyte and thus leading to lower OCV outputs. Figure 3h shows the calibration results, where the curve presents good linearity of the OCV outputs to the pH value with a *R* ^2^ of 0.998 and a sensitivity of 41.9 mV per decade of H^+^ concentration. The pH sensor also exhibits excellent anti‐interference ability and specificity for H^+^, as exhibited in Figure 3i, which has little OCV responses for these common electrolytes in bio‐fluid (like potassium chloride and ammonium chloride).

### 2.5 In vivo evaluation of the WDS for wireless wound exudate monitoring

**FIGURE 4:**
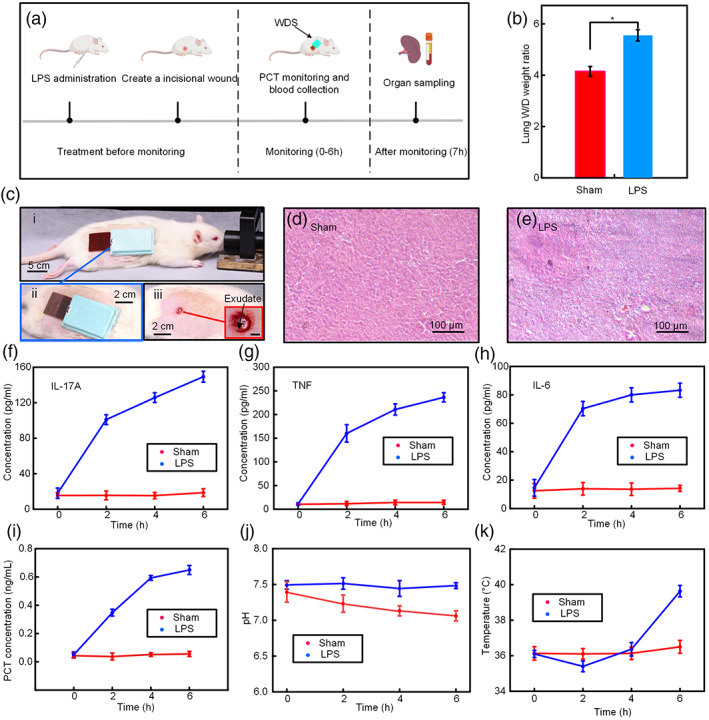
In vivo assessment of the performance of wound dressing system (WDS) for simultaneously monitoring of wound condition and sepsis diagnosis. (a) Experimental procedure for the LPS‐induced sepsis rat model. The procalcitonin (PCT) level, pH value, and skin temperature were recorded by WDS for 6 h. (b) The lung wet to dry weight ratio between the LPS group and sham group (*n* = 3). (c) Photograph of the WDS applied on the wound for exudate collection and wound condition monitoring (i), photograph of skin incision applied with (ii) and without (iii) WDS. Inset: enlarge view of circle skin incision. Scale bar: 0.25 cm. (d, e) Histological images for liver sections with hematoxylin and eosin staining from the sham group (d) and LPS group (e). (f–h) Pro‐inflammatory cytokine IL‐17A (f), TNF (g), and IL‐6 (h) level in the serum of mice in sham group and LPS group (*n* = 3). (i–k) Variation of PCT concentration, pH value in the wound exudate and skin temperature of mice from sham group and LPS group detected by WDS from 0 to 6 h (*n* = 3).

In order to evaluate the performance of the wireless and battery‐free WDS system in multiplex wound monitoring of sepsis relative parameters (PCT, pH, Temperature), a series of animal experiments were conducted. Figure 4a shows in vivo experimental procedure for LPS‐induced sepsis model. The SD rats (250 g, male) were first divided into two groups (LPS‐treated group, sham group). In the LPS‐treated group, the rats were received the injection of LPS (Sigma‐Aldrich, 20 mg/kg) and SD rat in sham group were intraperitoneal administrated same volume of PBS on the contrast. A circle skin incision (diameter: 0.5 cm, depth: 3 mm) was created on the back of each rat. The WDS was applied on the wound to monitor the variation of PCT, pH, and temperature between LPS‐treated group and sham group for 6 h (Figure 4c). Administration of LPS was commonly used to produce septic response which leads to damage of lung and liver in rat and mice.^33^, ^34^, ^35^ As shown in Figure 4b, the lung wet‐to‐dry weight ratio in LPS‐treated group is higher than sham group which indicated LPS cause an increasing of lung saturated.^36^ Histopathological analysis of liver (Figure 4d) was applied to substantiate the liver damage induced by LPS. The section stained with hematoxylin and eosin revealed typical liver injury (intercellular space increased, infiltration of inflammatory cell) compared with sham group. Meanwhile, the cytokine levels in the plasma of rat including IL‐17A, TNF, and IL‐6, the critical factors for the disorder of immune system and high mortality in sepsis model, were measured by flow cytometry analysis. The concentration of these pro‐inflammatory cytokines drastically increased after LPS administration at all the time (Figure 4f–h). The concentration of IL‐17A, TNF, and IL‐6 (149.9, 236.8, and 83.1 pg/ml) in the plasma at 6 h was obvious higher than sham group (13.2, 14.2, and 13 pg/ml) which indicated administration of LPS leads to a strong septic immune response in LPS‐treated group.^37^ The exudate fluidic secreted from wound consist of fluidic leaked out from blood vessel and composition of wound exudate is similar with the blood plasma.^38^ For wound monitoring, the PCT concentration, pH value, and temperature in the wound exudate in the LPS‐treated group and sham group were recorded by WDS per hour. As shown in Figure 4i, the PCT secreted dramatically after the stimulation of LPS and reach peak value at 6 h (0.6 ng/ml), on the contrast, there is no obvious secretion of PCT in sham group. As one of the representative biomarkers for septic disease diagnosis, the increasing PCT level reveals a high risk of sepsis due to systemic inflammation. In spite of PCT was diluted in exudate, the PCT sensor in WDS provides accuracy PCT concentration acquisition for wound exudate. In addition, the pH of the wound exudate in LPS‐treated group decreased from 7.7 to 7.3 (Figure 4j). An explanation for this result is SD rats with the sepsis may result in lactic acidosis which decreases the pH value in the serum.^39^, ^40^ Also, the temperature increasing of SD rat in LPS‐treated group caused by disorder of immune response was precisely recorded by temperature sensor in WDS. Compared with the more stable plot of temperature in sham group, the body temperature in LPS‐treated group was higher and reaches nearly 40°C at 6 h (Figure 4k). Therefore, we believe the WSD exhibits strong detection performance for septic relative parameters through wound exudate diagnosis.

## 3 CONCLUSIONS

In this work, we developed a wearable battery‐free and fully integrated WDS for timely wound monitoring and exudate management for early sepsis diagnosis. The fabricated device exhibits great flexibility which can applied on skin seamlessly and provide the potential to integrate with real dressing. The NFC‐based battery‐free soft electronic design enables wireless energy harvesting and data transmission. The soft silicone microfluidic‐based module can effectively avoid collect exudate sample and filtrate contamination during exudate collection. Key parameters in wound monitoring such as pH value, temperature, and sepsis‐related biomarker (PCT) can be precisely detected by electrochemical‐based biosensors in a wound monitoring patch. In vivo assessments show that the variation of pH value, temperature and PCT level can be continuously recorded, processed, and wirelessly transmitted to smartphone by NFC. Therefore, this wound management strategy provides a potential application prospect in wearable electronics and point of care diagnosis, especially in autoimmune disease, infectious disease, and tetanus.

## 4 METHODS

### 4.1 Fabrication of the flexible wireless sensing circuit

The circuit is manufactured by flexible printed circuit board processing techniques (as shown in Supplementary Table 3) of copper (thickness: 35 μm) with gold (thickness: 1 μm) plating on PI substrate. The entire fPCB measures just 50 mm × 30 mm by utilizing a simple two‐layer fPCB design (thickness: 2 mm, Supplementary Figure 4). The circuit is cover with PI protective layer on the surface to prevent short circuit. All components, including NFC receiver (ST25DV16K, STMicroelectronics), microcontroller (STM32L011F4, STMicroelectronics), potentiostat amplifier (AD8606, AD), and digital‐to‐analog converter (DAC8562, TI) are all soldered on fPCB using solder paste (CD‐LT 528, Conduction, China) at 138°C. The NFC antenna is designed to surround the rest of circuit components for the purpose of saving space. Then, the fabricated fPCB was put in the replica mold (Polyacrylate) fabricated by 3D printing equipment (HALOT‐SKY, CRE‐ALITY) and the surface was coated with 5 μm layer of Parylene C using special Parylene deposition equipment (PDS 2010 421 Labcoter2, Specialty Coating Systems Inc.) as isolation molecular layer. Then, the uncured Ecoflex (modulus: 82 kPa, Smooth‐on) mixed with green dye (6 wt%, Silcpig) was poured into the mold. After baking on a hot plate at 65°C for 20 min, the fPCB was then peel off from the mold.

### 4.2 Fabrication of the electrochemical sensors

The fabrication of electrochemical sensor begun with coating a 5 μm layer of Parylene C by chemical vapor deposition (CVD) using Parylene deposition equipment (PDS 2010 Labcoter2, Specialty Coating Systems Inc.), as the supporting layer of the gold. Then, a layer of Au with the thickness of 150 nm is deposited on the Parylene C layer by electron‐beam evaporation (E‐Beam, EBS‐500F, Junsun Tech Co., Ltd.). The Au layer was then spin‐coated (3000 rpm, 30 s) by AZ photoresist (5214, AZ Electronic Materials) and sent for baking at 110°C for 5 min. After UV exposure for 10 s with a mask of the pattern, the photoresist was developed in AZ 400K solution to remove unexposed photoresist for 3 min, followed by post‐baking at 110°C for 5 min. Finally, The Au is etched by the gold etchant (I2/KI solution, I2:KI:water = 1:4:40) and then rinsed in acetone to remove the remaining photoresist.

The PCT sensor is consisted with three electrodes. Therein, the graphene/Thi/PCT antibody modified gold electrode is the working electrode, Ag/AgCl modified electrode is the reference and the other gold electrode is the counter electrode. For the modification of working electrode, the mixing solution of graphene (2 mg/ml), Thi (2 mg/ml) and pyrenebutyric acid (PBA, 5 mM) were first prepared. Then, spaying 100 μl prepared solution on the working electrode on a 120°C hotplate with the help of a PI mask. The Ag/AgCl electrode was fabricated by screen‐printing Ag/AgCl ink on the gold electrode and then baking for 20 min on a 100°C hotplate. For the pH sensor, reference electrode was fabricated by the similar method with that of PCT sensor. Following, dropping 5 μl polyvinyl butyral (PVB) cocktail (79 mg PVB, 50 mg NaCl, and 1 mg block polymer PEO‐PPO‐PEO in 1 ml methanol solution) on the reference electrode. The sensing element on the working electrode was obtained by electrodepositing PANI film on the gold electrode with cyclic voltammetry from −0.1 to 1 V for 40 segments in a three‐electrode electrochemical system.

### 4.3 Fabrication of the microfluidic system

The integrated microfluidic system is fabricated based on soft lithography and replica method of PDMS.^41^ Briefly, the master for replica molding was fabricated by patterning SU‐8 (SU‐8 2015, Microchem) photoresists with a thickness of 50 μm on a 4‐in. silicon wafer. To reduce the adhesion during PDMS releasing procedure, the surface of mold master is treated by oxygen plasma treatment (energy: 5 kJ; Harrick plasma cleaner PDC‐002) for 1 min then salinized by depositing a molecular layer of trichloro (1H, 1H, 2H, 2H‐perfluoro‐octyl) silane (Sigma‐Aldrich). Next, the PDMS monomer (Sylgard‐184, Dow Corning) is mixed with the curing agent in a mass ratio of 15:1 to fabricate PDMS prepolymer. After degassing and in a vacuum environment for 15 min, the degassed PDMS prepolymer was applied onto the mold master with a thickness of 2 mm then sent for baking at 70°C for 30 min for cross‐linking of PDMS. We then chopped and peeled off the PDMS substrate from master mold, and then punch holes at the microchannel inlets and cut the extra PDMS at the end of the microchannel.

### 4.4 Surface modification of microfluidic system via the deposition of PVA

The hydrophilic treatment of PDMS substrate for wound exudate collection was performed by PVA surface modification.^42^ Briefly, the PDMS microfluidic system was treated by oxygen plasma treatment (energy: 5 kJ; Harrick plasma cleaner PDC‐002) for 1 min, follow by immersing into the 10% PVA solution (87% hydrolysis degree, Sigma‐Aldrich) for 20 min at room temperature. Then, the treated PDMS module was then sent to bake at 110°C for 15 min to remove residual PVA solution. Finally, a tape (magic tape, 3M) was attached on the top of the microchannel as shielding mask for further application.

### 4.5 Integration process for the flexible circuit, electrochemical sensors and microfluidic model

Integration process for WDS begun with the covalently binding between PDMS microfluidic system and electrochemical sensors. Briefly, the top surface of PH sensor was tightly attached with a tap as shielding mask; then the aminopropyltriethoxysilane (Thermo Scientific) was deposited on the uncover area in the sensor layer for adhesion promotion. Both of electrochemical sensor layer and PVA‐treated PDMS microfluidic system were sent for oxygen plasma treatment (energy: 5 kJ; 1 min). Next, the shielding masks cover on the sensor layer and microfluidic system were removed before covalently binding and the device was then baked at 80°C for 30 min. After baking, the electrochemical sensors integrated with microfluidic chip was connected by PCB connector (five pins, TE connectivity) and stored in a dry cabinet for further application.

### 4.6 The filtration performance measurement of microfluidic system

To investigate the filtration performance, the microsphere with different diameters (1.2, 6, 10.2, and 18 μm) were injected into microfluidic chip via a syringe pump with the flow rate of 10 μl/min. We counted the number of microspheres filtrated by microfilter and number of microspheres escaped through the outlet. The filtrating rate is considered as the percentage of the number of filtrated droplets compared to the total number of droplets flowing in the microfluidic chip.

### 4.7 Flow simulation

Computational studies are investigated using COMSOL Multiphysics (COMSOL, Burlington, MA). The three‐dimensional models are constructed and analyzed for the flow characteristics of wound exudate collected in the microfluidic system which bending under 60°. The flow rate of wound exudate in the collection chamber and streamline around microfilter were profiled. Considering that the inlet liquid flow rate and outlet pressure of models are defined as 10 μl/min and atmospheric pressure, respectively, which corresponding to the lower Reynolds number. We applied a laminar flow model for solving the Navier–Stokes equations.^43^

### 4.8 Animal experiments

All animal experiments were performed in accordance with City University of Hong Kong regulations (project license number A‐0664). The SD rat (weight: 250 g, male) were purchased from Chinese University of Hong Kong. Before the LPS‐induce procedure, the SD rats were acclimatized for 1 week. To establish the LPS‐induced sepsis rat model, six rats were divided equally into two groups as LPS‐induced group and sham group. The SD rats in LPS‐induced group and sham group were administrated with LPS (15 mg/kg) and PBS (same volume with LPS‐induced group) by intraperitoneal injection respectively. Then, the hair on back of the rats was shaved. After the sterilization of skin, a circle skin incision with the diameter of 0.5 cm were created by puncher for two groups. Next, the WDS was applied on skin incision to monitor wound situation including temperature, PCT secretion and PH per hour for 6 h. At the same time, the 20 μl of blood was collected per hour via tail vein for cytokine secretion profiling. After monitoring, the animals with different treatments (*n* = 3) were killed, and their lung and liver were harvested. The lung wet‐to‐dry (*W*/*D*) weight ratio was measured. Hematoxylin and eosin staining was carried out for liver histological examinations.

### 4.9 Cytokine measurement by flow cytometry

The blood sample was first injected through a nylon mesh filter with porous size of 70 μm to isolate immune cell. Then, red blood cells were lysed using ACK buffer (Thermo Scientific) then washed with PBS. A commercial mouse inflammatory cytokine kit (BD Biosciences, CA) was chosen to quantify cytokine secretion in the blood samples (the detection mechanism of cytokine using flow cytometry technology as shown in Supplementary Figure 5). The treated blood sample were mixed the microbeads and incubated with a phycoerythrin (PE)‐conjugated (emission wavelength: 488 nm) detection antibody reagent for 3 h at room temperature. After removing the unbind PE molecules by for improving the signal‐to background ratio, the blood sample was sent for analysis of flow cytometry (Accuri C6 Plus, BD Biosciences). Gating strategies were set based on fluorescence minus one sample. The fluorescence intensity of a targeted cytokine was measured, which then was converted to the cytokine level by using a calibration curve between the bead fluorescence intensities and known concentrations of each selected cytokine (Supplementary Figure 5).

### 4.10 Statistics

Standard errors in plots are represented by error bars. The Student's two‐tailed, unpaired *t* test was adopted to compare two groups with the corresponding *p*‐value. Each asterisk in a plot represents a significant difference between two data groups (*p* < 0.05), unless additionally specified in the figure caption.

## AUTHOR CONTRIBUTIONS

**Jiyu Li:** Conceptualization (lead); data curation (lead); investigation (lead); methodology (equal); project administration (lead); software (equal); writing – original draft (lead); writing – review and editing (equal). **Xingcan Huang:** Data curation (equal); formal analysis (equal); investigation (lead); methodology (lead); writing – original draft (supporting). **Yawen Yang:** Data curation (supporting); formal analysis (supporting); investigation (lead); methodology (equal); software (equal); visualization (equal); writing – original draft (equal). **Jingkun Zhou:** Investigation (equal); software (equal); writing – review and editing (equal). **Kuanming Yao:** Methodology (supporting); visualization (supporting). **Jian Li:** Investigation (supporting); resources (supporting). **Yingying Zhou:** Investigation (supporting); resources (supporting). **Meixi Li:** Writing – review and editing (supporting). **Tsz Hung Wong:** Software (supporting). **Xinge Yu:** Funding acquisition (lead); resources (lead); supervision (lead); writing – review and editing (equal).

## CONFLICT OF INTEREST

The authors declare no competing conflict of interests.

### PEER REVIEW

The peer review history for this article is available at https://publons.com/publon/10.1002/btm2.10445.

## Supporting information


**SUPPLEMENTARY FIGURE 1** Photograph of top side (i) and back side (ii) of fPCB (a) before and after soldering with electronics
**SUPPLEMENTARY FIGURE 2** Experiment setup for the measurement of peel force between microfluidic ship and flexible sensing patch.
**SUPPLEMENTARY FIGURE 3** Simulated driving pressure of fluidic went through the microfiltration in vertical (i) and horizontal (ii) cross section.
**SUPPLEMENTARY FIGURE 4** Schematic design of the wearable and battery‐free wound dressing system.
**SUPPLEMENTARY FIGURE 5** Working principle of cytokine detection mechanism using flow cytometry.
**SUPPLEMENTARY FIGURE 6** Calibration curves for IL‐6, IL‐17A and TNF in the serum measured by flow cytometry analysis.
**SUPPLEMENTARY TABLE 1** Temperature sensor characteristics.
**SUPPLEMENTARY TABLE 2**. General operating conditions for the temperature sensor in the MCU
**SUPPLEMENTARY TABLE 3**. Manufacturing process of flexible printed circuit using photolithographic technology.

## Data Availability

All data needed to evaluate the conclusions in the papers are present in the paper and/or the Supplementary Information.
