# Supplementary material for: Wearable and battery‐free wound dressing system for wireless and early sepsis diagnosis
Source: Bioeng Transl Med. 2023 Feb 1;8(6):e10445. doi: 10.1002/btm2.10445 (PMC10658553; doi:10.1002/btm2.10445)
Supplement: Supplementary file 1 — SUPPLEMENTARY FIGURE 1 Photograph of top side (i) and back side (ii) of fPCB (a) before and after soldering with electronics SUPPLEMENTARY FIGURE 2 Experiment setup for the measurement of peel force between microfluidic ship and flexible sensing patch. SUPPLEMENTARY FIGURE 3 Simulated driving pressure of fluidic went through the microfiltration in vertical (i) and horizontal (ii) cross section. SUPPLEMENTARY FIGURE 4 Schematic design of the wearable and battery‐free wound dressing system. SUPPLEMENTARY FIGURE 5 Working principle of cytokine detection mechanism using flow cytometry. SUPPLEMENTARY FIGURE 6 Calibration curves for IL‐6, IL‐17A and TNF in the serum measured by flow cytometry analysis. SUPPLEMENTARY TABLE 1 Temperature sensor characteristics. SUPPLEMENTARY TABLE 2. General operating conditions for the temperature sensor in the MCU SUPPLEMENTARY TABLE 3. Manufacturing process of flexible printed circuit using photolithographic technology. [file BTM2-8-e10445-s001.docx]

**Supplementary Information**

# Wearable and battery-free wound dressing system for wireless and early sepsis diagnosis

Jiyu Li ^a, b†^, Xingcan Huang^a†^, Yawen Yang^a†^, Jingkun Zhou ^a,b†^, Kuanming Yao ^a^, Jian Li ^a ,b^, Yingying Zhou ^c^, Meixi Li^d^, Tsz Hung Wong ^a^ , Xinge Yu^a,b*^

^a^ Department of Biomedical Engineering, City University of Hong Kong, Kowloong Tong, Hong Kong

^b^ Hong Kong Center for Cerebra-Cardiovascular Health Engineering, Hong Kong Science Park, New Territories 999077, Hong Kong

^c^ Department of Biomedical Engineering, Hong Kong Polytechnic University, Kowloon, Hong Kong

^d^ Leshan Hospital of Traditional Chinese Medicine, Leshan, Sichuan Province 614000, China

† These authors contributed equally to this work.

Corresponding author: Xinge Yu, [xingeyu@cityu.edu.hk](mailto:xingeyu@cityu.edu.hk)


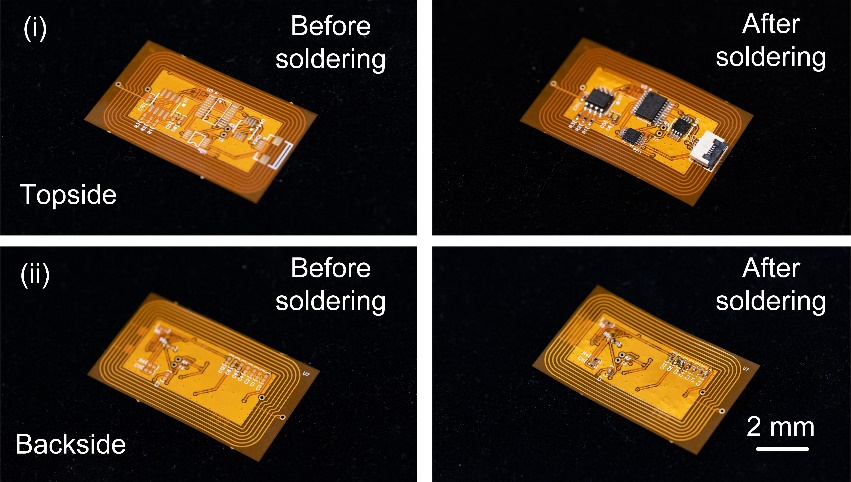


Supplementary Figure 1| Photograph of top side (i) and back side (ii) of fPCB (a) before and after soldering with electronics


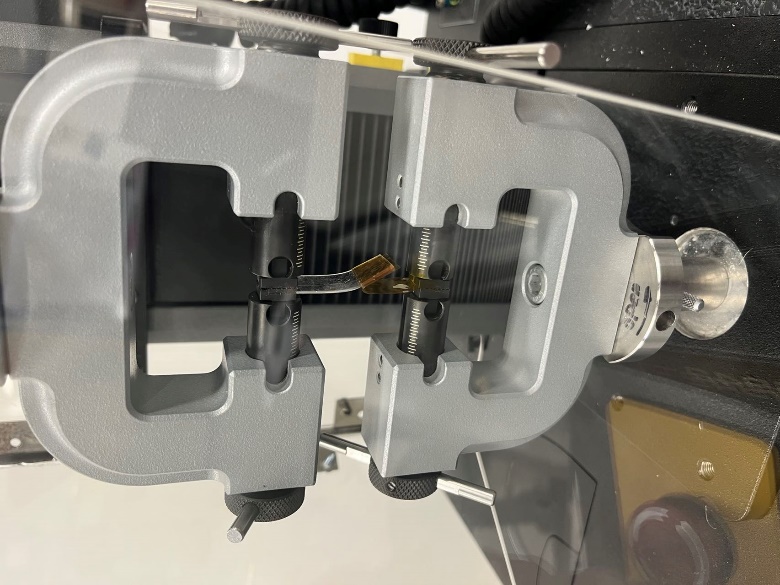


Supplementary Figure 2|Experiment setup for the measurement of peel force between microfluidic ship

and flexible sensing patch.


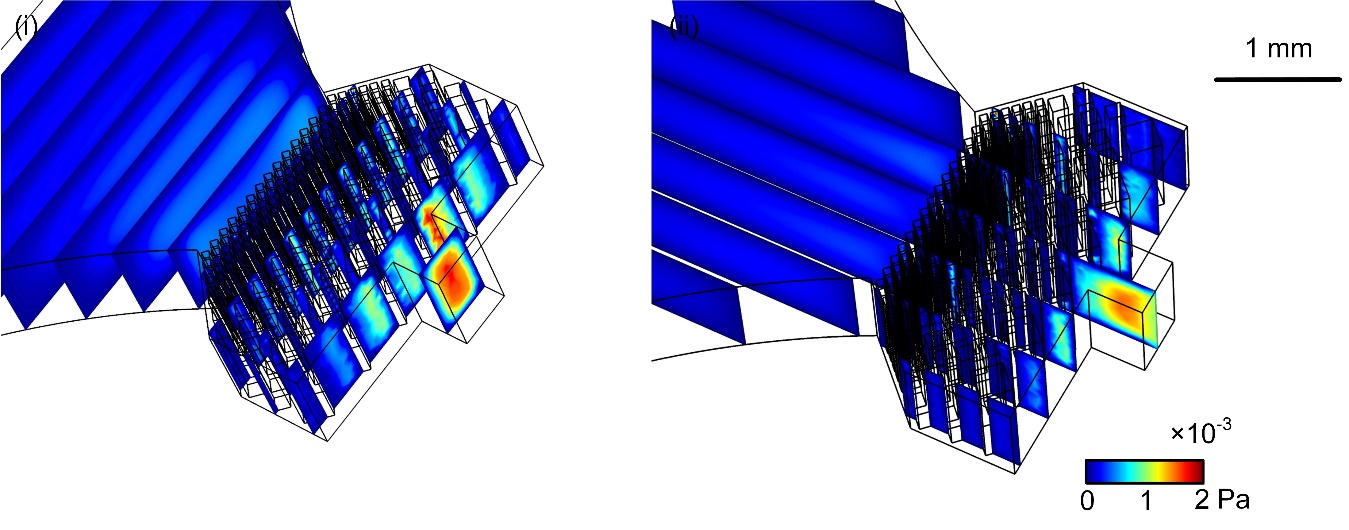


Supplementary Figure 3| Simulated driving pressure of fluidic went through the microfiltration in

vertical (i) and horizontal (ii) cross section.


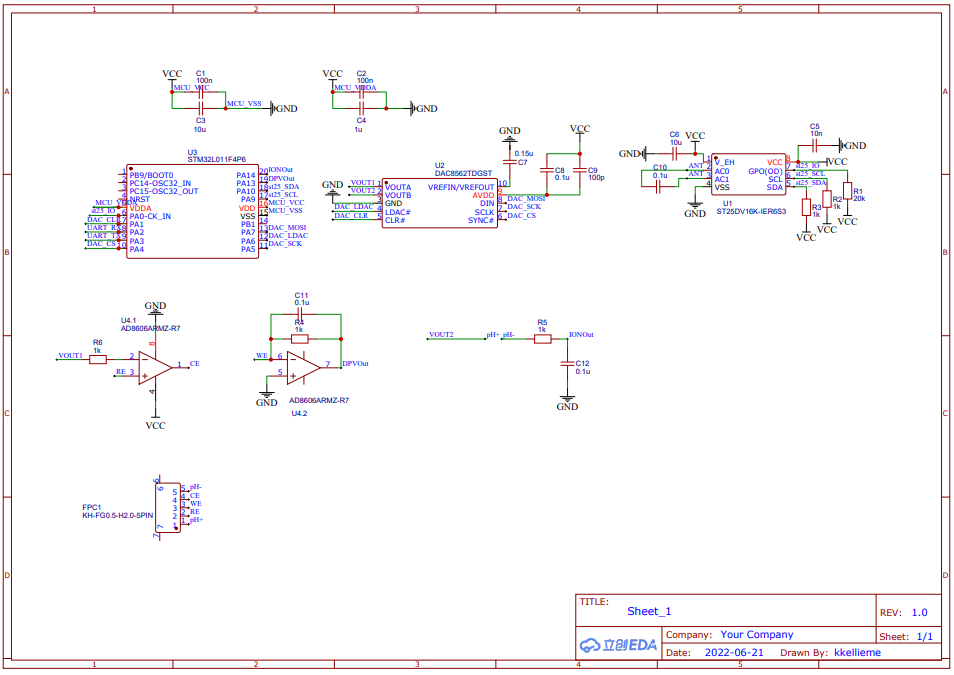


Supplementary Figure 4| Schematic design of the wearable and battery-free wound dressing system.


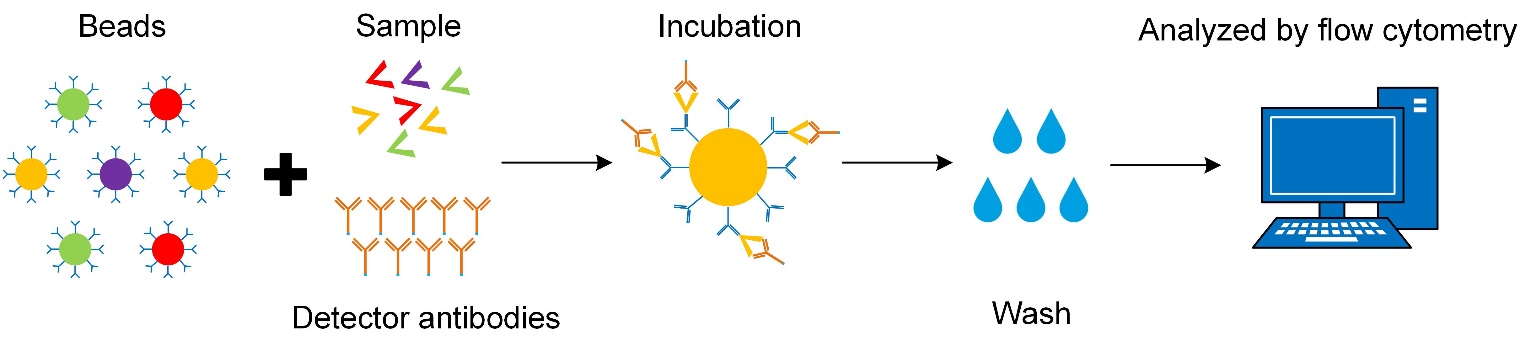


Supplementary Figure 5| Working principle of cytokine detection mechanism using flow cytometry.


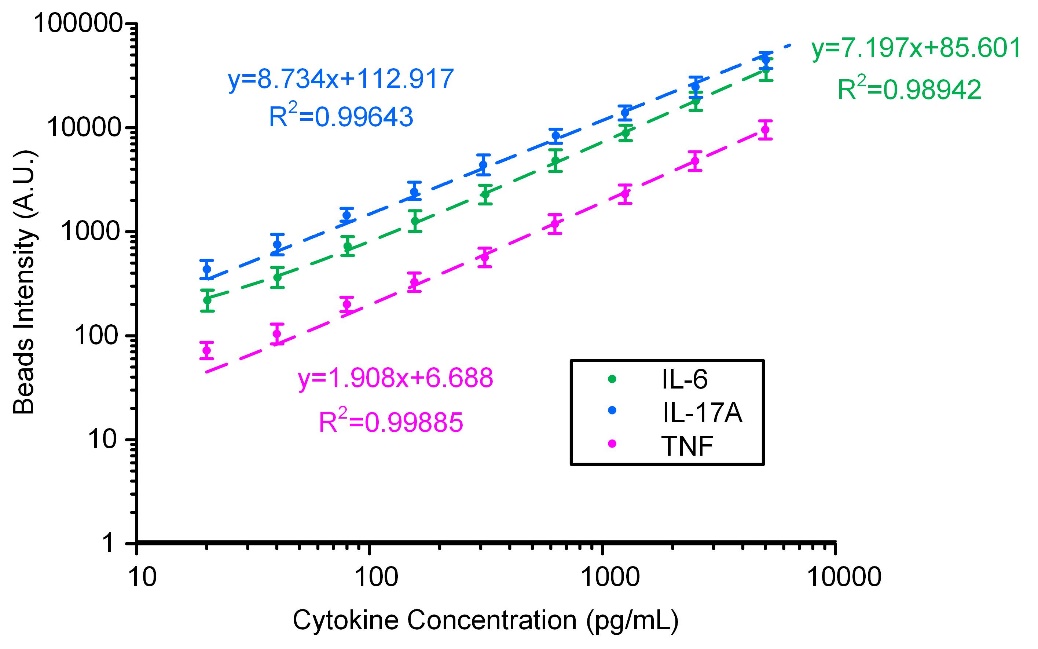


Supplementary Figure 6| Calibration curves for IL-6, IL-17A and TNF in the serum measured by flow

cytometry analysis.

| Symbol | Parameter | Conditions | Min | Max | Unit |
| --- | --- | --- | --- | --- | --- |
| T_A_ | Temperature range | Maximum power  dissipation (Range A) | -40 | 85 | °C |
|  |  | Maximum power  Dissipation (Range B) | -40 | 105 |  |
|  |  | Maximum power  Dissipation (Range C) | -40 | 125 |  |
| T_J_ | Junction temperature range  (Range A) | -40 °C ≤ TA ≤ 85°C | -40 | 105 |  |
|  | Junction temperature range  (Range B) | -40 °C ≤ TA ≤ 105°C | -40 | 125 |  |
|  | Junction temperature range  (Range C) | -40 °C ≤ TA ≤ 125°C | -40 | 130 |  |

Supplementary table 1| Temperature sensor characteristics.

| Symbol | Parameter | Min | Typ | Max | Unit |
| --- | --- | --- | --- | --- | --- |
| T_L_ | VSENSE linearity with temperature | - | ±1 | ±2 | °C |
| Avg_Slope | Average slope | 1.48 | 1.61 | 1.75 | mV/°C |
| V_130_ | Voltage at 130°C±5°C^(2)^ | 640 | 670 | 700 | mV |
| I_DDA(TEMP)_ | Current consumption | - | 3.4 | 6 | uA |
| t_START_ | Startup time | - | - | 10 | uS |
| T_S_temp_ | ADC sampling time when reading the temperature | 10 | - | - |  |

Supplementary table 2|. General operating conditions for the temperature sensor in the MCU

| **Step** | **Manufacturing method** | **Description** |
| --- | --- | --- |
| 1. | Shearing | To optimize the material utilization, cut the material as working size for different design and layout concern. |
| 2. | Drilling | To fit customers’ design and process demand, drill different holes on boards, such as alignment holes, testing vias, components holes, etc |
| 3. | Plating through hole | Since both the top and bottom layer are not conductive, plate copper on the hole-wall after drilling to realize pattern connection between layers. This process applies to the making of double-sided flexible boards. |
| 4 | Dry film lamination | To create an etching resist layer, pass pre-treated boards through hot roller to laminate dry film on it. |
| 5 | Exposure | Transfer the designed circuit pattern image to dry film laminated on the foil to create an exposure area. |
| 6 | Development | Cast UV (ultraviolet) light on the dry film, the dry film on the exposure area will be polymerized and hardened, and the dry film on non-exposure area can be removed and expose copper. |
| 7 | Etching | After development, non-exposure area will be moved away by chemical reaction and the protected area will be remained. |
| 8 | Stripping | Remove the dry film covering the circuit pattern and make pattern exposed. |
| 9 | Optical inspection | Use AOI tester to check the quality of circuit. |
| 10 | Pre-lamination | The board will be covered with insulating layer called ‘coverlay’ to protect the circuit. |
| 11 | Hot press | The board with coverlay pass through hot press machine to form adhesion between copper and coverlay. |
| 12 | Metal finish | Coat the bonding area with nickel or gold to protect and keep the terminator’s function. |
| 13 | Screen printing | If there’re needs to print visible information like logos or numbers onto the boards, silk screening will be applied. |

Supplementary table 3|. Manufacturing process of flexible printed circuit using photolithographic technology.
